# Optimierung der mikrobiellen Diagnostik durch Einführung einer Standard Operating Procedure „Blutkulturen“ in der zentralen Notaufnahme

**DOI:** 10.1007/s00063-020-00729-5

**Published:** 2020-10-02

**Authors:** H. M. Orth, S. Al Agha, M. Kempe, C. Mackenzie, M. Michael, M. Bernhard, B. -E. O. Jensen

**Affiliations:** 1 Klinik für Gastroenterologie, Hepatologie und Infektiologie, Universitätsklinikum Düsseldorf, Düsseldorf, Deutschland; 2 Antibiotic Stewardship (ABS) Team, Universitätsklinikum Düsseldorf, Düsseldorf, Deutschland; 3 Zentrale Notaufnahme, Universitätsklinikum Düsseldorf, Moorenstraße 5, 40225 Düsseldorf, Deutschland; 4 Zentralapotheke, Universitätsklinikum Düsseldorf, Düsseldorf, Deutschland; 5 Institiut für Medizinische Mikrobiologie und Krankenhaushygiene, Universitätsklinikum Düsseldorf, Düsseldorf, Deutschland

**Keywords:** Sepsis, Infektion, Mikrobielle Diagnostik, Antiinfektive Therapie, Blutkulturen, Infection, Sepsis, Microbial diagnostics, Anti-infective treatment, Blood cultures

## Abstract

**Einleitung:**

Zentrale Notaufnahmen stellen die Eintrittspforte für viele stationär aufzunehmende Patienten in einem Krankenhaus dar und sind häufig der Ausgangspunkt für die antiinfektive Diagnostik und Therapie von Notfallpatienten. In dieser retrospektiven Untersuchung soll der Frage nachgegangen werden, wie die Etablierung einer Standard Operating Procedure (SOP) „Blutkulturen“ und deren Schulung die mikrobielle Diagnostik in einer zentralen Notaufnahme verbessern kann.

**Methodik:**

In einer Vorher-und-nachher-Untersuchung wurde über einen jeweils 3‑monatigen Zeitraum (11/2017 bis 01/2018 und 11/2018 bis 01/2019) die Anzahl der abgenommenen Blutkulturen, die Rate an Blutkulturen/1000 Fälle, die Anzahl positiver Blutkulturen und die Häufigkeit typischer Hautkeime analysiert. Im Zeitraum zwischen den evaluierten Zeitabschnitten wurde eine SOP „Blutkulturen“ in Zusammenarbeit mit dem Antibiotic-Stewardship(ABS)-Teams und der zentrale Notaufnahme entwickelt, implementiert und geschult. Ein positives Votum der Ethikkommission der Heinrich-Heine-Universität (2019-392-RetroDEuA) lag vor.

**Ergebnisse:**

Die pflegerischen und ärztlichen Mitarbeiter wurden zu 92 % bzw. 93 % geschult. Die Anzahl der abgenommenen Blutkulturen stieg von 1757 auf 2872 um 64 % ebenso wie die Anzahl der Blutkulturen/1000 Fälle von 287 auf 481 (68 %). Die Anzahl der positiven Blutkulturen reduzierte sich von 18,6 auf 13,7 % (*p* < 0,05). Typische Hautkeime fanden sich in 34,4 % und 26,4 % der Fälle (*p* < 0,05).

**Interpretation:**

Die durch Schulungen begleitete Einführung einer SOP „Blutkulturen“ in der zentralen Notaufnahme kann einen relevanten Beitrag zur antimikrobiellen Diagnostik leisten und sowohl die Quantität als auch die Qualität erhöhen.

## Abstract (en)

**Introduction:**

The emergency department (ED) is the main port of entry for patients with infectious diseases, the place where a number of diagnostic procedures are performed and treatment is often initiated. The aim of this retrospective study was to estimate the influence of the establishment and introduction of a blood culture standard operating procedure (BC-SOP) and of the subsequent training of microbial diagnostics in an ED.

**Methods:**

In a before and after study over a study period of 3 months each (November 2017–January 2018 and November 2018–January 2019), the number of blood cultures taken, the rate of blood cultures per 1000 patients, the number of positive blood cultures and the frequency of typical skin pathogens were evaluated. In the interim time between the two study periods, a BC-SOP was developed in collaboration with the hospital’s antibiotic stewardship team and subsequently introduced with staff training in the ED. The study was approved by the local ethics committee of the medical faculty of the Heinrich Heine University (2019-392-RetroDEuA).

**Results:**

In total 92% of the nursing personnel and 93% of the medical personnel received training. The total number of blood cultures increased from 1757 to 2872 (64% increase) and the rate of blood cultures per 1000 patients from 287 to 481 (68% increase). The number of positive blood cultures decreased from 18.6% to 13.7% (*p* < 0.05). Typical skin pathogens were found in 34.4% and 26.4% of the cases, respectively (*p* < 0.05).

**Conclusion:**

The development, introduction and training of a BC-SOP in the ED can make a relevant contribution to the microbial diagnostics and increase the quantity as well as the quality.

## Einleitung

Die zentrale Notaufnahme stellt eine wichtige Nahtstelle zwischen der ambulanten und stationären Notfallversorgung dar und ist häufig Ausgangspunkt für die Identifikation von gefährdeten Patienten mit Infektionen in einem Krankenhaus [5]. In der zentralen Notaufnahme sind daher besondere Anforderungen an die Identifikation von Patienten mit Infektionen, die entsprechende Diagnostik und den Beginn einer antiinfektiven Therapie zu stellen [1, 2, 6, 7]. Die zentrale Notaufnahme eignet sich daher auch in besonderem Maß, um Optimierungsmaßnahmen in der mikrobiellen Diagnostik umzusetzen.

Auch wenn das Ergebnis dieser Untersuchung nicht unmittelbar in der zentralen Notaufnahme verfügbar ist, gehört die Abnahme von Blutkulturen gemäß den internationalen Sepsisleitlinien zu den Basismaßnahmen im Rahmen der Versorgung von Patienten mit potenziell schwerwiegenden Infektionen und ist daher auch Teil des 1‑Stunden-Bündels der Surviving Sepsis Campaign [2]. Blutkulturen sollten daher vor dem Beginn einer antibiotischen Therapie unter antiseptischen Bedingungen noch in der zentralen Notaufnahme gewonnen werden [6]. In den vergangenen Jahren wiesen Studien aber immer wieder eine schlechte Qualität und Quantität der Blutkulturdiagnostik in Notaufnahmen nach [3, 4]. Falsch-positive Befunde durch mit Hautkeimen kontaminierte Blutkulturen können mit einer nichtindizierten Verwendung von Antibiotika einhergehen und die Krankenhauskosten erhöhen [8].

Vor diesem Hintergrund ist es Aufgabe des Antibiotic-Stewardship(ABS)-Teams eines Krankenhauses, zusammen mit dem Team aus der zentralen Notaufnahme einen Maßnahmenkatalog zur Verbesserung der Qualität und Quantität der Blutkulturdiagnostik zu erarbeiten und umzusetzen. Nach Bool et al. [3] sollen entsprechende Maßnahmen 1) Schulungen und Feedback („education and feedback“), 2) die Etablierung von Maßnahmenbündeln/Standard Operating Procedures (SOP, „bundled approaches“) und 3) Technik und Material („technique and equipment“) enthalten. Eine Optimierung der Qualität und Quantität der Blutkulturdiagnostik stellt damit einen wesentlichen Beitrag zur Qualitätssteigerung der mikrobiellen Diagnostik und damit auch der antiinfektiven Therapie dar.

Ziel der vorliegenden retrospektiven Untersuchung war es daher zu prüfen, inwieweit die Entwicklung, Einführung und Schulung einer SOP „Blutkulturen“ in einer zentralen Notaufnahme die Qualitäts- und Quantitätsindikatoren für die Blutkulturdiagnostik verbessern können.

## Material und Methodik

Im Rahmen des vorliegenden Projektes wurden die folgenden 5 Punkte durchgeführt:

1. Entwicklung einer SOP „Blutkulturen“ durch das ABS-Team und die zentrale Notaufnahme;
2. Einführung eines Blutentnahmeadapters mit Blutkulturhalter in der zentralen Notaufnahme;
3. Schulung in der Anwendung der SOP „Blutkulturen“ und der fachgerechten Abnahme von Blutkulturen in der zentralen Notaufnahme;
4. retrospektiver Vergleich von Prozessindikatoren der Blutkulturdiagnostik in der zentralen Notaufnahme;
5. Punktprävalenzanalyse in der zentralen Notaufnahme.

Die retrospektive Studie wurde durch die Ethikkommission der Heinrich-Heine-Universität (Studiennummer: 2019-392-Retro DEuA) genehmigt.

### SOP „Blutkulturen“

**Figure 1:**
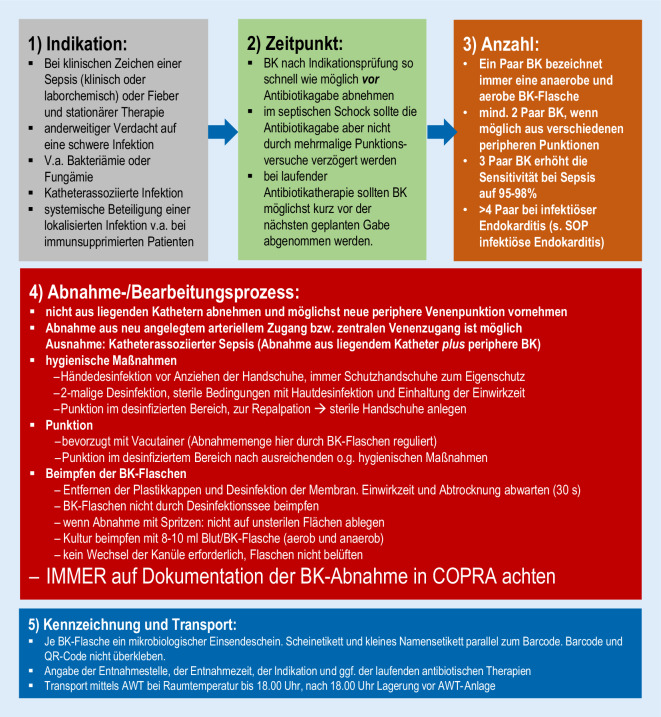

Durch das ABS-Team des Universitätsklinikums Düsseldorf wurde in Zusammenarbeit mit Mitarbeiterinnen und Mitarbeitern der zentralen Notaufnahme ein Konzept zur Optimierung der Quantität und Qualität in Form einer SOP „Blutkulturen“ entwickelt (Abb. 1). Bekannte Vorgaben für die Abnahme von Blutkulturen wurden berücksichtigt und in die SOP „Blutkulturen“ integriert [1–4, 8].

### Schulungsmaßnahmen

Durch die Schulungsmaßnahmen des pflegerischen und ärztlichen Personals der Zentralen Notaufnahme des Universitätsklinikums Düsseldorf sollte durch das ABS-Team die Indikationsstellung zur Blutkulturdiagnostik, die Durchführung der Blutkulturabnahme und die weitere Präanalytik standardisiert vermittelt werden. Hierzu erfolgte ein mündlicher Vortrag mit der Dauer von rund 15  min und einer Gruppengröße von etwa 5 Personen in der zentralen Notaufnahme. Die Anwesenheit wurde mittels Listen erfasst. Der Inhalt der Schulung umfasste die Erläuterung der Bedeutung der Blutkulturdiagnostik, die Aushändigung und Erläuterung der zugrunde liegenden SOP „Blutkulturen“ sowie die Demonstration der Durchführung der Abnahme von Blutkulturen an einem Dummy. Abgerundet wurden die Schulungsmaßnahmen durch die Möglichkeit, Fragen zu stellen und Unklarheiten zu beseitigen.

### Blutabnahmeadapter

Vor Umsetzung des Maßnahmenbündels waren Blutkulturen mit einer Standard-20  ml-Spritze mit folgender Injektion in 2 Blutkulturflaschen abgenommen worden. Gleichzeitig mit den Schulungsmaßnahmen wurde dann durch die Einführung eines neuen Blutentnahmesystems zur vereinfachten Asservierung von Blutkulturen eine Maßnahme zur Reduktion von Kontaminationsmöglichkeiten installiert. Für die Blutentnahme wurde das VACUETTE®-System um einen Blutkulturhalter (Greiner Bio-One GmbH, Frickenhausen, Deutschland, Artikel-Nr. 450181) ergänzt. Die fachgerechte Anwendung des Blutentnahmeadapters für die Blutkulturen wurden im Rahmen der Schulungsmaßnahmen vermittelt.

### Erfassung von Qualitäts- und Quantitätsindikatoren

Um die Wirksamkeit der Maßnahmen zu evaluieren, wurden routinemäßig bestimmte Qualitäts- und Quantitätsindikatoren in der nachfolgenden Vor-und-nachher-Untersuchung gegenübergestellt. Folgende Prozessindikatoren wurden im Rahmen der vorbeschriebenen Qualitätssicherungsmaßnahmen erfasst:

- Rate abgenommener Blutkulturen/1000 Patienten;
- Anzahl positiver Blutkulturen;
- Anzahl mutmaßlicher Kontamination in den positiven Blutkulturen (retrospektive Ermittlung bei Nachweis von Hautkeimen – koagulasenegative Staphylokokken, *Cutibacterium acnes*, coryneforme Bakterien – anhand der beim jeweiligen Patienten gestellten Diagnose).

### Punktprävalenzanalyse

Zur Prüfung einer adäquaten Indikationsstellung zur Blutkulturdiagnostik wurde an jeweils einem Tag der 2 Studienzeiträume eine Punktprävalenzerfassung durchgeführt. Dabei wurde überprüft, in welchem Umfang bei den am jeweiligen Tag in der zentralen Notaufnahme behandelten Patienten mit Vorliegen entsprechender Symptome eine Blutkulturdiagnostik initiiert wurde, die den Kriterien der SOP Blutkulturdiagnostik entsprach.

### Zeitlicher Verlauf

Im Zeitraum zwischen 01.09.2018 und 31.10.2018 erfolgten die Schulungsmaßnahmen und die Einführung der SOP Blutkulturdiagnostik. Am 01.11.2018 erfolgte der offizielle Start der Implementierung der SOP „Blutkulturen“ in der zentralen Notaufnahme. Als postinterventioneller Erfassungszeitraum wurde der Bereich vom 01.11.2018 bis 31.01.2019 gewählt. Als Vergleichszeitraum wurde der Vorjahreszeitraum vom 01.11.2017 bis 31.01.2018 gewählt, um den Einfluss einer Saisonalität (z. B. von Atemwegsinfektionen) zu reduzieren.

Die Punktprävalenzerfassung der korrekten Indikationsstellung zur Blutkulturdiagnostik erfolgte am 15.11.2017 und am 15.11.2018.

### Patientenkollektiv

Zur Fallzahlplanung diente die Anzahl der nichttraumatologischen Patienten in der zentralen Notaufnahme in den genannten Zeiträumen. Bei den verarbeiteten Daten handelte es sich nicht um individuelle Patientendaten, sondern um Qualitätsindikatoren aus diagnostischen Ergebnissen, die auf die Gesamtzahl der behandelten Patienten in den Überprüfungszeiträumen bezogen wurden. Die Daten geben Aufschluss über die diagnostische Qualität im Gesamtkollektiv der behandelten Patienten. Zum Zweck der Qualitätskontrolle erfolgte eine primäre Erfassung der Daten ohne eine individuelle Aufklärung oder Einwilligung. Die errechneten Indikatoren lassen keinen Rückschluss auf individuelle Befunde zu. Es existierte kein Zuordnungsschlüssel, da eine Zuordnung technisch aus den ermittelten Qualitätsindikatoren nicht möglich war. Die Vorgaben der Datenschutzgrundverordnung (DSGVO) wurden eingehalten.

### Statistische Analyse

Zur statistischen Auswertung wurden definierte Merkmale verglichen. Die Angaben der Daten erfolgten als Absolutzahl oder als prozentualer Anteil. Unterschiede zwischen den Patientenkollektiven wurde mittels χ^2^-Test analysiert. Eine Fehlerwahrscheinlichkeit von *p* < 0,05 wurde als statistisch signifikant gewertet.

## Ergebnisse

**Figure 2:**
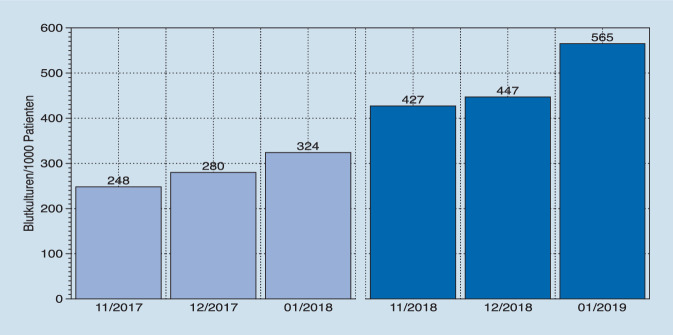

**Figure 3:**
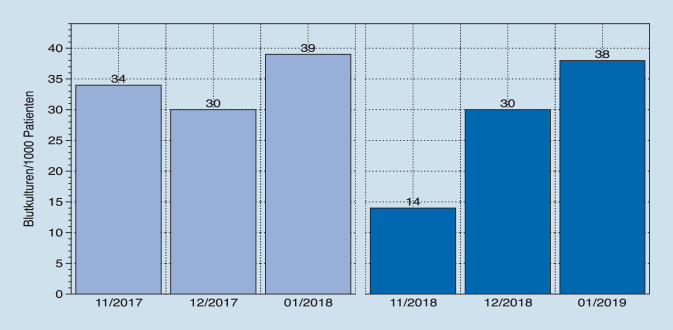

Die Schulungsmaßnahme erreichte 48 von 52 pflegerischen Mitarbeitern (92 %) und 26 von 28 ärztlichen Mitarbeitern (93 %). Die Anzahl der abgenommenen Blutkulturen in dem jeweiligen Zeitraum 11/2017 bis 01/2018 und 11/2018 bis 01/2019 hat sich von 1757 auf 2872 erhöht (Tab. 1). Die Anzahl der abgenommen Blutkulturen/1000 Fälle von 287 auf 481 hat sich nahezu verdoppelt (Abb. 2). Die Anzahl positiver Blutkulturen sank im Untersuchungszeitraum von 18,6 % (326) auf 13,7 % (394, *p* < 0,05). Ebenfalls konnte der Anteil typischer Hauptkeime von 38,7 % (126) auf 31,7 % (129) reduziert werden (*p* < 0,05). In der retrospektiven Auswertung der Patientenakten wurde festgestellt, dass bei einem Teil der Patienten die nachgewiesenen Hautkeime mutmaßlich doch Ursache der Infektkonstellation waren, so in 11,1 % (*n* = 3) im Vergleichszeitraum und in 19,4 % (*n* = 6) des postinterventionellen Erfassungszeitraums (*p* < 0,05; Tab. 1). Der Anteil typischer Hauptkeime ohne plausiblen Fokus als Marker einer mutmaßlichen Kontamination sank somit von 34,4 % (*n* = 112) auf 26,4 % (*n* = 104, *p* < 0,05, Abb. 3).

**Table 1:** 

	*Untersuchungszeitraum 11/17 bis 01/18* Vor Einführung der SOP „Blutkulturen“	*Untersuchungszeitraum 11/2018 bis 01/2019* Nach Einführung der SOP „Blutkulturen“
*Anzahl abgenommene Blutkulturen*	*1757*	*2872*
Aerob	864	1430
Anaerob	892	1442
Nicht definiert	1	0
Anzahl Blutkulturen/1000 Fälle, *n*	287	481
*Anzahl positiver Blutkulturen, n* (%)	*326 (18,6* *%)*	*394 (13,7* *%)*
Davon typische Hautkeime, *n* (%)	126 (38,7 %)	129 (32,7 %)
Davon plausibler Foci (lt. Patientenakte, *n* (%)	14 (11,1 %), 3 Pat. Einmal Katheterinfekt Einmal inf. Shuntaneurysma Einmal Portinfekt	25 (19,4 %), 6 Pat. 4‑mal Portinfekt Einmal Demers-Katheter-Infekt Einmal Gangrän bei AVK
Typische Hautkeime ohne plausiblen Focus (Kontaminationsrate, *n* (%)	112 (34,4 %)	104 (26,4 %)

*SOP* Standard Operating Procedure, *n* Anzahl, *Pat.* Patienten, *inf.* infiziert, *AVK* Arterielle Verschlusskrankheit

### Prävalenzuntersuchungen

Zu den 2 definierten Zeitpunkten am 15.11.2017 und 15.11.2018 wurden 94 bzw. 105 Patienten in der zentralen Notaufnahme behandelt. Gemäß den Definitionen der SOP „Blutkulturen“ in der zentralen Notaufnahme ergab sich bei 7 Patienten im ersten Zeitraum und 5 Patienten im zweiten Zeitraum gemäß der SOP „Blutkulturen“ die Indikation zur Blutkulturdiagnostik. In allen Fällen und Situationen wurden Blutkulturen bei bestehender Indikation durch die Mitarbeiter abgenommen (Erfolgsrate: 100 %). Die korrekte Anzahl abgenommener Blutkulturflaschen gemäß der Definition der SOP „Blutkultur“ in der zentralen Notaufnahme war jedoch im ersten Zeitraum mit 86 % höher als im zweiten Zeitraum mit 60 %.

## Diskussion

Die Blutkulturdiagnostik zählt zu den wesentlichen Maßnahmen einer mikrobiellen Diagnostik in der zentralen Notaufnahme bei potenziellen Patienten mit einer Infektion [2, 3]. Deshalb ist die Abnahme von Blutkulturen eine der Basismaßnahmen im Rahmen einer Sepsisbehandlung und wurde 2018 auch in das 1‑Stunden-Bündel der Surviving Sepsis Campaign integriert [2]. Dies erfolgt auch, obwohl die Ergebnisse der Blutkulturdiagnostik nicht unmittelbar in der zentralen Notaufnahme vorliegen [2]; die so gewonnenen mikrobiellen Erkenntnisse können aber für die weitere stationäre Therapie und die Auswahl eines geeigneten Antiinfektivums entscheidend sein [1].

ABS-Teams helfen unter anderem, durch regelmäßige Visiten auf Stationen einen rationalen Umgang mit Antiinfektiva herbeizuführen. Ergänzend unterstützen ABS-Teams aber auch in der geeigneten Auswahl an mikrobieller Probengewinnung und im Umgang mit Blutkulturen als Diagnostikum im realen Stationsalltag [9]. Vor diesem Hintergrund wurde es als gemeinsame Zielsetzung des ABS-Teams und der Zentralen Notaufnahme des Universitätsklinikums Düsseldorf angesehen, die Quantität und Qualität der Blutkulturdiagnostik am eigenen Standort zu verbessern.

Mehrere Schritte sind dabei notwendig, um entsprechende Maßnahmen effektiv umzusetzen [3]: 1) Schulungen und Feedback („education and feedback“), 2) die Etablierung von Bündeln/Standard Operating Procedures („bundled approaches“), und 3) Technik und Material („technique and equipment“). Entsprechend diesen Vorgaben wurden folgende Maßnahmen initialisiert: 1) Durch Schulungsmaßnahmen konnten über 90 % der pflegerischen und ärztlichen Mitarbeiter der zentralen Notaufnahme geschult werden. 2) Dies erfolgte, nachdem interdisziplinär durch das ABS-Team und Mitarbeiterinnen und Mitarbeiter der zentralen Notaufnahme eine übersichtliche SOP „Blutkulturen“ entwickelt worden war. 3) Um auch hinsichtlich technischer und materieller Aspekte eine Optimierung herbeizuführen, wurde ein Blutentnahmeadapter eingeführt, der ein direktes Anschließen der Blutkulturflaschen an die Punktionskanüle ermöglicht und damit kontaminations- und verletzungsträchtige Arbeitsschritte wie ein Umfüllen des abgenommenen Bluts obsolet macht.

Die ausgeführten Maßnahmen hatten sowohl hinsichtlich der Qualitäts- als aus auch Quantitätsindikatoren positive Auswirkungen: Die Anzahl der durchgeführten Blutkulturen pro 1000 Fälle stieg nach Intervention um 86 % von 287 auf 481. Die Rate an Hautkeimen ohne plausiblen Fokus sank im ersten Monat nach Intervention von 34 % auf 14 %, stieg aber im weiteren Verlauf auf das Ausgangsniveau wieder an. Dies verdeutlicht das nur transiente Wirken von Schulungsmaßnahmen, die in regelmäßigen Abständen wiederholt werden sollten. Der Anteil korrekt durchgeführter Indikationsstellungen konnte laut Punktprävalenzanalyse nicht weiter gesteigert werden, hier wurde in beiden Fällen eine Erfolgsrate von 100 % erreicht.

Aufgrund der geringen Fallzahlen sind jedoch insbesondere die Ergebnisse der Punktprävalenzerfassung mit großer Vorsicht zu interpretieren und nur mit Zurückhaltung zu bewerten. Darüber hinaus muss beachtet werden, dass insbesondere die Anzahl an falsch-positiven Befunden und damit an Kontaminationen im zweiten 3‑monatigen Untersuchungsintervall wieder fast auf das Ausgangsniveau anstieg. Dies kann ein starker Hinweis darauf sein, dass Schulungsmaßnahmen hinsichtlich der Hygiene bei der Abnahme von Blutkulturen regelmäßig fortgesetzt werden müssen.

Besonders positiv könnte sich die Auswahl der zentralen Notaufnahme als Umsetzungslokalisation für qualitätssteigernde Maßnahmen bei der mikrobiellen Diagnostik aber vor allem daher erweisen, da regelmäßig eine Vielzahl an Rotationsassistenten aus verschiedenen Kliniken und Fachabteilungen hier tätig ist und diese die erlernten Informationen zur Blutkulturdiagnostik zu einem späteren Zeitpunkt in ihre eigenen Kliniken und Abteilungen transportieren können und somit als Multiplikatoren dienen.

### Limitationen

In der vorliegenden retrospektiven Untersuchung wurden folgende Punkte nicht erfasst: der korrekte Zeitpunkt der Blutkulturdiagnostik (Abnahme der Blutkulturen vor oder nach Antibiotikaerstgabe) sowie die Korrektheit der Befüllvolumina der Blutkulturflaschen. Der Anteil der Wirksamkeit von Schulungsmaßnahmen und neuen Blutabnahmesystemen konnte aufgrund der Gleichzeitigkeit der Einführung nicht differenziert beziffert werden. Die geringe Diskrepanz der Anzahl aerober und anaerober Blutkulturflaschen (Tab. 1) deutet auf eine in Einzelfällen unvollständige Blutkulturabnahme hin. Möglicherweise lag diesen Fällen eine Dislokation der Punktionskanülen zugrunde, diese Annahme kann jedoch retrospektiv nicht überprüft werden.

Die pflegerische und ärztliche Besetzung in der zentralen Notaufnahme unterliegt einer hohen Fluktuation, einerseits durch Stellenwechsel, andererseits durch Rotationswechsel in der Weiterbildung. Vor diesem Hintergrund ist es sehr wahrscheinlich, dass sich in beiden retrospektiven Untersuchungszeiträumen die personellen Besetzungen unterschieden haben. Die Auswirkung dieser Personalfluktuation kann an dieser Stelle nicht bestimmt werden, die Einführung der SOP soll zukünftig diesen Einfluss aber reduzieren.

Die Folgeeffekte der Maßnahmen können quantitativ reduzierte und qualitativ bessere, weil für den korrekten Erreger angepasste, Antibiotikatherapien sein und somit positiven Einfluss auf Nebenwirkungen, Liegezeiten, Behandlungsergebnisse und Kosteneffektivität haben. Dies wurde aber in der vorliegenden Untersuchung nicht spezifisch erfasst.

## Fazit für die Praxis

Eine in Zusammenarbeit zwischen ABS-Team und zentraler Notaufnahme entwickelte, geschulte und eingeführte SOP „Blutkulturen“ kann unter Verbesserung der materiellen Rahmenbedingungen (Blutentnahmeadapter) zur Optimierung der Blutkulturdiagnostik führen und sowohl die Quantität als auch die Qualität erhöhen.
